# Recent Progress in Metal‐Organic Frameworks for Applications in Electrocatalytic and Photocatalytic Water Splitting

**DOI:** 10.1002/advs.201600371

**Published:** 2017-01-13

**Authors:** Wei Wang, Xiaomin Xu, Wei Zhou, Zongping Shao

**Affiliations:** ^1^ Department of Chemical EngineeringCurtin UniversityPerthWA6845Australia; ^2^ Jiangsu National Synergetic Innovation Center for Advanced Materials (SICAM)State Key Laboratory of Materials‐Oriented Chemical EngineeringCollege of Chemical EngineeringNanjing Tech University (NanjingTech)Nanjing210009P. R. China; ^3^ Jiangsu National Synergetic Innovation Center for Advanced Materials (SICAM)State Key Laboratory of Materials‐Oriented Chemical EngineeringSchool of Energy Science and EngineeringNanjing Tech University (NanjingTech)Nanjing210009P. R. China

**Keywords:** catalysis, hydrogen evolution reaction, metal‐organic frameworks, oxygen evolution reaction, water splitting

## Abstract

The development of clean and renewable energy materials as alternatives to fossil fuels is foreseen as a potential solution to the crucial problems of environmental pollution and energy shortages. Hydrogen is an ideal energy material for the future, and water splitting using solar/electrical energy is one way to generate hydrogen. Metal‐organic frameworks (MOFs) are a class of porous materials with unique properties that have received rapidly growing attention in recent years for applications in water splitting due to their remarkable design flexibility, ultra‐large surface‐to‐volume ratios and tunable pore channels. This review focuses on recent progress in the application of MOFs in electrocatalytic and photocatalytic water splitting for hydrogen generation, including both oxygen and hydrogen evolution. It starts with the fundamentals of electrocatalytic and photocatalytic water splitting and the related factors to determine the catalytic activity. The recent progress in the exploitation of MOFs for water splitting is then summarized, and strategies for designing MOF‐based catalysts for electrocatalytic and photocatalytic water splitting are presented. Finally, major challenges in the field of water splitting are highlighted, and some perspectives of MOF‐based catalysts for water splitting are proposed.

## 1 Introduction

The global demand for energy has increased rapidly and continuously in recent decades due to the quickly expanding human population and industrialization; as a result, there has been a significant increase in the utilization of traditional fossil fuels, which has caused severe environmental problems, such as the greenhouse effect, air pollution and water pollution. The quickly expanded energy consumption has resulted in major concerns about energy crises due to the limited fossil fuels resources. For a sustainable future, the development of alternative energy material that is clean and sustainable is highly desirable but remains a major challenge. Among the various energy carriers (materials), hydrogen is one of the most ideal and cleanest energy materials for the future due to its high gravimetric energy density (120 vs. 44 MJ kg^−1^ for gasoline), high combustion efficiency, non‐toxicity, clean exhaust products, and renewable and storable nature. During the past two decades, tremendous attention has been given to the field of hydrogen energy by researchers and governments around the world. However, the success of the hydrogen economy is strongly determined by the availability of useful routes for the large‐scale generation of hydrogen. Currently, the production of hydrogen mainly relies on steam reforming and partial oxidation of fossil fuels (natural gas or other hydrocarbons), causing concerns about serious CO_2_ emissions and limited natural resources.^1^, ^2^, ^3^ Water, one of the most abundant resources on earth, is composed of hydrogen and oxygen atoms. Water splitting is one of the most effective ways to produce hydrogen. Among the various routes for hydrogen generation from water at low temperature, direct water splitting using solar/electrical energy over photocatalysts/electrocatalysts is highly promising because of its sustainability.^4^, ^5^, ^6^, ^7^, ^8^, ^9^

Water splitting (H_2_O → H_2_ + 1/2O_2_) consists of two half reactions, known as the oxygen evolution reaction (OER) and the hydrogen evolution reaction (HER). However, these reactions have sluggish kinetics and require catalysts. In the electrochemical process, the OER and the HER are generally catalyzed by precious metal (Ir/Ru and Pt, respectively) materials to achieve favorable reaction kinetics.^10^, ^11^, ^12^, ^13^ Unfortunately, noble metals suffer from low abundance and high cost, hindering their large‐scale use in water electrolysis. To ensure sustainable hydrogen production, it is of great importance to seek earth‐abundant alternatives to precious metal‐based catalysts with excellent activity and robust stability.^14^, ^15^, ^16^, ^17^, ^18^, ^19^, ^20^, ^21^, ^22^ For example, several electrocatalysts composed of earth‐abundant elements (e.g., Fe, Co and Ni) were found to be promising alternatives to precious catalysts, achieving high OER and HER activity.^14^, ^15^, ^16^, ^17^, ^18^ In addition, some carbon‐based or heteroatom‐doped carbon materials have been evaluated as innovative options as electrocatalysts for the OER and HER.^19^, ^20^, ^21^, ^22^ The availability of different carbons (nanotubes, graphene, etc.) with adjustable compositions has markedly increased the number of candidates for OER/HER electrocatalysis.^23^, ^24^, ^25^ Solar‐driven H_2_ generation from water using semiconductor‐based photocatalysts is another attractive route to solve the energy and environmental problems.^26^, ^27^, ^28^, ^29^, ^30^ To date, a number of metal oxide‐based photocatalysts have been demonstrated to be effective for water splitting under UV light irradiation.^31^, ^32^, ^33^ In particular, TiO_2_ has been reported as a benchmark for the UV‐light‐driven water splitting reactions due to its good photo‐stability, low toxicity, large abundance and low cost. Unfortunately, TiO_2_ has a large band gap of 3.2 eV, which can only be used in the UV light range, which includes only 5% of all solar energy (solar conversion efficiency in UV light is only 2% compared with 16% when visible light up to 600 nm can be utilized). Thus, the development of new photocatalysts with high photocatalytic activity under visible light irradiation is one of the most attractive research topics in photocatalytic water splitting.^34^, ^35^, ^36^, ^37^ In addition to the material composition, the activity of catalysts for electrocatalytic/photocatalytic water splitting relies heavily on the morphology of the catalyst.^38^, ^39^, ^40^, ^41^ Thus, optimizing the catalyst composition and morphological structure is of critical importance to achieve highly efficient hydrogen production from water splitting.

Metal‐organic frameworks (MOFs) are a new class of porous materials with unique electronic, optical and catalytic properties.^42^, ^43^ In addition, they can be used as precursors for the fabrication of various metal, metal oxide‐carbon composites or pure carbon materials with rich morphological structures and versatile properties.^43^ In applications as electrocatalysts or photocatalysts or their precursors, MOFs offer several advantages, such as high design flexibility, tunable pore channels, large surface‐to‐volume ratios, flexibility to be functionalized with various ligands and metal centers, and rich compositions.^43^ The metal centers separated by organic linkers in MOFs can be considered as quantum dots; consequently, short diffusion lengths of the charge carriers can be achieved during the electrocatalytic and photocatalytic reactions.^44^ The specific surface areas and band gaps of MOFs can be tailored by tuning the organic ligands and/or metal centers, so their electrocatalytic and photocatalytic activities can be tailored to maximize their performance. In recent years, MOFs have been exploited directly as photocatalysts or as their precursors for hydrogen generation from water splitting, the degradation of organic pollutants and the reduction of CO_2_ into useful fuels.^45^, ^46^, ^47^, ^48^, ^49^ Recently, MOF‐based materials have also proved to be particularly suitable for electrocatalytic water splitting.^50^, ^51^, ^52^ In the last five years, tremendous efforts have been made to apply MOFs as photocatalysts and electrocatalysts for water splitting, and interest in this research field is projected to continue increasing. Thus, a review of the recent advances and challenges of MOF‐based materials in photocatalytic and electrocatalytic water splitting is highly desirable.

Herein, the recent development of MOF‐based materials for electrocatalytic and photocatalytic water splitting reactions is presented. Several critical factors that determine the activity for water splitting reactions are summarized, and strategies related to the design of catalysts are emphasized. Major challenges in the fields of photocatalytic and electrocatalytic water splitting are highlighted, and some perspectives from the current progress in the development of MOF‐based catalysts are given. Directions of the future research are also presented, with emphasis on achieving the desired MOF functionality and establishing structure‐property relationships to identify and rationalize the factors that determine the catalytic performance. This paper aims to provide a comprehensive review of the recent progress in this dynamic field, as well as some guidelines for the further development of highly efficient photocatalysts and electrocatalysts based on MOFs for water splitting.

## 2 Fundamentals of Water Splitting Reactions

### 2.1 Electrocatalytic Water Splitting

#### 2.1.1 Basic Principles

Electrocatalytic water splitting involves two half reactions (OER and HER), and the mechanistic schemes of the OER and HER have been proposed in the literature.^53^, ^54^, ^55^, ^56^ The OER, which is a four‐electron process, is more complex than the HER and involves several surface‐adsorbed intermediates. In the following section, we mainly focus on the mechanistic study of the OER while that of the HER is described only briefly.

In the HER, the chemical adsorption and desorption of H atoms are competitive processes. A good HER catalyst should have a bond with the adsorbed H* (the asterisk indicates a bond to the catalyst surface) that is sufficiently strong to enable the proton‐electron‐transfer process and also sufficiently weak to ensure easy bond breaking and release of the produced H_2_ gas.^53^ The change in the Gibbs free energy for H* adsorption on an electrocatalyst surface (Δ$G_{H}^{*}$) can be applied to evaluate both H* adsorption and H_2_ desorption using the HER free energy diagram.^54^ The optimal Δ$G_{H}^{*}$ should be zero, under which condition the HER reaches the maximum rate.^53^ More importantly, a “volcano curve” correlation has been proposed between the experimental HER activity (HER exchange current density) and the quantum chemistry‐derived Δ$G_{H}^{*}$ for various catalyst surfaces.^54^ As a result, the relationship between the nature of the electrocatalyst surface and the HER kinetics can be established.

The OER pathways, in acidic or alkaline media, include elementary steps that differ according to different mechanisms, yet all involve the adsorption/desorption of intermediates, such as HO*, O* and HOO*.^55^, ^56^, ^57^ The free adsorption energies of the OER intermediates at selected potentials on Pt (111) and Au (111) and some other metals were studied in acidic environment by Rossmeisl et al. using density functional theory (DFT) calculations.^58^ The most difficult step in the OER is the formation of HOO* on the metal surface by splitting water on an adsorbed oxygen atom (O*). This step is downhill in free energy at high electrode potentials. At lower potentials, although water can dissociate to O*, the OER is initiated only on the oxidized surface, which makes this step slower than the O* formation process. In other words, the formation of OOH* from O* is uphill for the OER at the equilibrium potential of 1.23 V vs. reversible hydrogen electrode (RHE). Applying a voltage to move the potential positively away from 1.23 V (the difference defined as the overpotential) is thus necessary for spontaneous OER. The calculations show that the OER on Pt and Au surfaces should start at approximately 1.8 V. Simple linear relations between the stability of different intermediates and OER activity were found when the analysis was extended to other metals, which suggests that the oxygen adsorption energy is a good descriptor of the capability of a metal‐based electrocatalyst for the OER.^58^

In addition to metallic catalysts, the OER mechanism on oxide catalysts has also been studied using computational methods.^59^ Rossmeisl and co‐workers investigated the trends in the electrocatalytic properties of the most stable (110) surfaces of RuO_2_, IrO_2_ and TiO_2_. Similar to the findings on metal surfaces, the binding energies of O*, HO* and HOO* on the (110) surfaces of these rutile oxides showed universal linear relations. Based on this, a volcano plot was constructed to describe the trends in OER activity according to a simplified descriptor, the O* binding energy. It was found that RuO_2_ binds oxygen slightly too weakly, while IrO_2_ binds oxygen too strongly, leading to a higher overpotential, which was also observed in experiments.^60^ However, TiO_2_ binds O* too weakly, and it displays a low OER activity. These results suggest that a material that binds oxygen slightly more strongly than RuO_2_ is expected to exhibit even better OER activity.

The origin of the overpotential for OER catalysis was also studied using DFT calculations on various oxides.^61^ A universal scaling relation between the binding energies of the HOO* and HO* intermediates was identified, which defined the lowest theoretical overpotential for the OER on oxide surfaces. This led to a general description of OER activity with the introduction of a single descriptor (Δ*G*_O*_−Δ*G*_HO*_). For the oxides considered, the OER activity could not be greatly enhanced beyond RuO_2_ by tailoring the binding between the intermediates and the oxide surface. To avoid the limitations defined by the universal scaling relationship, relative stabilization of HOO* compared to HO* must be achieved. In this regard, three‐dimensional (3D) structures are likely to stabilize HOO*.

#### 2.1.2 Factors to Determine the Electrocatalytic Activity

Generally, the catalytic activity of an electrocatalyst for water splitting is determined by the intrinsic activity and the number of active sites. For oxide‐based electrocatalysts, the intrinsic activity is often related to the material composition, mixed valence states of the compositional cations (redox couples), crystal structure, metal‐oxygen bond energy, oxygen vacancy concentration, electronic conductivity and charge transfer capability.^56^, ^62^, ^63^, ^64^, ^65^ The number of active sites can be increased by building high‐surface‐area structures, tuning the morphology and creating nanostructured catalytic systems. Compositing with other catalytic materials or conductive supports can result in hybrids with enhanced activity and more active sites, which is sometimes known as the synergistic effect. The most effective methods to maximize the HER/OER activity include tailoring the surface and/or bulk properties (by the selection of cations and anions), optimizing the morphology (by the use of advanced synthetic procedures), enhancing the charge transfer process (by the functional modification of the surface electronic structure) and forming composite or hybrid catalysts. These methods may produce more active sites for HER/OER and ideal pathways for the transportation of reactants and gaseous products (i.e., hydrogen and oxygen). The strategies for enhancing electrocatalytic activity are not limited to oxides and can be, in principle, applied to other types of electrocatalysts. Additionally, researchers often take advantage of several combined strategies to improve the efficiency of electrocatalysts in the HER/OER.

The morphology and microstructure are crucial characteristics for electrocatalysts because they have a direct correlation with the number of active sites and, therefore, the catalytic activity.^66^, ^67^ For example, a simple self‐template strategy was developed to fabricate hollow Co‐based bimetallic sulfide (M_x_Co_3−x_S_4_, M = Zn, Ni and Cu) polyhedra from homogenous bimetallic MOFs.^66^ The combination of polyhedral morphology, hollow structure, homo‐incorporation of a second metal element and high Brunauer‐Emmett‐Teller (BET) surface area significantly enhanced the HER activity of Co_3_S_4_. The hollow Zn_0.30_Co_2.70_S_4_ exhibited the highest catalytic activity among the four electrocatalysts, indicating that cation selection is very important to achieve high electrocatalytic activity for water splitting.

In addition, nanostructured electrocatalysts generally benefit from increased specific surface area and, therefore, have more active sites for the electrocatalysis, which can be tailored by the preparation methods and annealing conditions.^68^, ^69^ For example, Shi et al. utilized an in situ carburization method to prepare MoC encapsulated by a graphitized carbon shell (nanoMoC@GS) electrocatalyst from a Mo‐based MOF.^68^ The nanoMoC@GS showed favorable activity in acidic media as an electrocatalyst for HER, which stemmed from the synergistic effects of the ultrafine MoC, ultrathin and conductive GS, high porosity and high surface area.^68^

Other methods to improve the surface area, such as synthesizing nanoparticles (NPs) and combining NPs with high‐surface‐area supports, have been used to enhance the activity of electrocatalysts for water splitting.^70^, ^71^ For example, Li et al. synthesized a nitrogen‐doped Fe/Fe_3_C@graphitic layer/carbon nanotube hybrid (Fe/Fe_3_C@NGL‐NCNT) using MIL‐101 (Fe) MOF as the precursor.^70^ This Fe/Fe_3_C@NGL‐NCNT hybrid showed superior OER activity and stability compared with the commercial Pt/C, which may originate from the abundant active sites and the synergistic effect of the unique architecture.

The charge transfer capability is also essential for achieving high electrocatalytic activity for water splitting, and the coupling of some functional materials, such as reduced graphene oxide (RGO), to MOF‐based electrocatalysts can improve the charge transfer capability (conductivity).^72^, ^73^ For example, Tang et al. used a simple pyrolyzing method to synthesize a porous Mo‐based hybrid from a polyoxometalate‐based MOF and graphene oxide (POMOFs/GO), which showed improved performance for the HER.^73^

### 2.2 Photocatalytic Water Splitting

#### 2.2.1 Mechanism and Reaction Steps

Studies on splitting water into hydrogen and oxygen using light (photons) originated from the discovery of the Honda‐Fujishima effect in 1967. Water splitting using photocatalysts has since been widely investigated.^74^, ^75^, ^76^, ^77^, ^78^ Previous reviews of water splitting using semiconductors as photocatalysts have demonstrated the basic principles of the water splitting process.^76^, ^77^, ^78^ The electrons in the valence band (VB) of the photocatalyst are transferred to the conduction band (CB), and holes are left in the VB after absorbing UV and/or visible light, creating electron‐hole pairs. The photogenerated electron‐hole pairs can induce redox reactions similar to water electrolysis. Specifically, water molecules are reduced by the electrons to generate H_2_ and are oxidized by the holes to produce O_2_, completing the water splitting reactions.

Water splitting into H_2_ and O_2_ is an energetically uphill reaction with a standard Gibbs free energy change (Δ*G*) of +237 kJ mol^−1^ (corresponding to 1.23 eV). Therefore, the band gap of the photocatalysts and the edges of the CB and VB must be suitable for water splitting. The bottom level of the CB should be more negative than the redox potential of H^+^/H_2_ (0.0 V vs. normal hydrogen electrode (NHE)), while the top level of the VB should be more positive than the redox potential of O_2_/H_2_O (1.23 V vs. NHE).^76^ The theoretical minimum band gap for water splitting is therefore 1.23 eV, which is equivalent to a light wavelength of approximately 1100 nm. However, not all semiconductors meet the requirements for water splitting. For metal oxide‐based photocatalysts, the VB mainly consists of O 2p orbitals, and the top level of the VB is much higher than 1.23 V vs. NHE. Therefore, the oxidation‐reduction potentials (ORPs) of O_2_/H_2_O and H^+^/H_2_ are positioned between the top level of the VB and the bottom level of the CB. Higher photon energy than the band gap of the photocatalyst is needed due to an activation barrier in the charge transfer process in the water splitting reactions. At the same time, the much wider band gaps of these materials make them only photoactive in UV light. As approximately 50% of the solar spectrum consists of visible photons (400 < λ < 800 nm), it is critical to develop active photocatalysts with high activity under visible light for photocatalytic water splitting.

The development of photocatalysts is very important to enable water splitting with visible light. The main steps in the photocatalytic water splitting reactions should be tailored to meet the requirements for photocatalysts capable of water splitting. There are three steps in the photocatalytic water splitting reactions, which has been demonstrated in some informative reviews.^76^, ^77^, ^78^ The first step is the formation of electron‐hole pairs by incident photons. When the energy of incident light is greater than the band gap energy, the electrons in the VBs can be excited and transferred into the CB. Meanwhile, holes are generated in the VB. However, the band structure is only a thermodynamic requirement. Other factors, such as charge separation, mobility and the lifetime of photogenerated electrons and holes can also affect the photocatalytic activity for water splitting. The second step is charge separation and diffusion to the catalyst surface without recombination of the photogenerated carriers, which is drastically affected by the crystal structure, particle size and crystallinity of the photocatalyst.^79^, ^80^ A higher crystallinity can lead to superior charge migration efficiency because the defects in a photocatalyst with lower crystallinity act as recombination centers for the photogenerated electron‐hole pairs, which decreases the photocatalytic activity. A smaller particle size of the photocatalyst also suppresses the possibility of electron‐hole pair recombination. The final step is the reduction and oxidation of surface‐adsorbed species by the photogenerated electrons and holes to generate H_2_ and O_2_, respectively. In this step, the surface active sites of the photocatalyst play vital roles in efficient water splitting. Co‐catalysts, such as Pt, are usually loaded onto the photocatalyst surface as active sites to reduce the activation energy for the HER.^81^ These processes affect the overall efficiency of water splitting based on a semiconductor‐based photocatalyst.

#### 2.2.2 Factors to Determine the Photocatalytic Activity

The main factors that determine the photocatalytic activity of the photocatalysts for water splitting include the band gap energy/visible light absorption capability, active sites/co‐catalysts and charge transfer/separation efficiency. Covalent modification is a method to reduce the band gap energy of photocatalysts. For example, the band gap of MOFs can be reduced by a diazo coupling with amino‐substituted ligands and other molecules.^82^ The photocatalytic activity of these modified MOFs corresponds to a red shift of the absorption edge, suggesting that the band gap energy/visible light absorption capability plays a vital role in the photocatalytic activity.

The incorporation of active co‐catalysts is an effective way to increase the number of active sites for water splitting.^83^, ^84^, ^85^, ^86^, ^87^ For example, the well‐defined cages of MIL‐101(Cr) MOF were used to engage the molecules of a high‐valent di‐*µ*‐oxo dimanganese catalyst with high activity for photo‐electrochemical (PEC) water oxidation and the incorporation of MnTD ([(terpy)Mn(*µ*‐O)_2_Mn](terpy)]^3+^; terpy: 2,2′:6′,2′′‐terpyridine) improved the turnover number of MIL‐101(Cr) more than 20‐fold while maintaining the initial high rate in the PEC water oxidation reaction.^83^ In another study, Hansen and Das found that MnTD⊂MIL‐101(Cr) showed superior activity to MIL‐101(Cr) and MnO_2_ catalysts for the OER.^84^ These studies suggested that the incorporation of active co‐catalysts can greatly enhance the photocatalytic activity for water splitting, and the selection and incorporation method of the co‐catalysts should be optimized.

The charge transfer/separation efficiency of the photocatalyst plays a critical role in photocatalytic water splitting. The construction of heterojunctions is an effective way to enhance the charge transfer/separation capability of electron‐hole pairs.^88^, ^89^ For example, a MOF‐derived Co_3_O_4_/TiO_2_ composite photocatalyst with 2 wt.% Co loading and a p‐n heterojunction, exhibited a much higher hydrogen evolution rate than the conventional Co_3_O_4_/TiO_2_ nanocomposite (≈ 7‐fold enhancement).^88^

The photocatalytic activity of MOF‐based photocatalysts is determined by several crucial factors, such as the band gap energy, active sites/co‐catalyst and charge transfer capability. These factors are often closely related. For example, an azo‐carboxylic acid can be used as an organic linker to construct a Gd‐based MOF with a reduced band gap.^90^ Gd‐MOF has high photocatalytic activity for the HER due to its high visible light absorption capability. The addition of Ag co‐catalyst improved the HER activity of Gd‐MOF by providing more active sites and improving the charge transfer capability.

## 3 Recent Advances in MOF‐Based Catalysts for Water Splitting

Because of the many outstanding features of MOFs, such as tunable pore channels, high specific surface area, easy tailoring of the material composition, rich morphological structure, and capability to act as precursors for the preparation of various metal/metal oxide/carbon composites and carbon materials of various properties, during the past five years, the applications of MOFs as catalysts or the precursors of catalysts for electrocatalytic and photocatalytic water splitting reactions for hydrogen generation have been extensively exploited. Both the direct application of MOFs for water splitting and application as a precursor for metal/metal oxide/carbon composites or porous carbon materials (by leaching of the metal/metal oxide from the composites), which were then applied as electrocatalysts or photocatalysts, have been reported. Additionally, MOFs were studied as catalysts for both the OER and HER, and the reactions were conducted in acidic and alkaline electrolytes.

### 3.1 Electrocatalytic Water Splitting

For electrocatalytic water splitting, the direct application of MOFs as electrocatalysts was first reported in 2011 by Nohra et al., who pioneered the use of polyoxometalate‐based MOFs (POMOFs) for the HER.^91^ The structural properties were investigated but their electrocatalytic activity was only briefly studied and the efficiency of POMOFs to replace Pt catalyst for the HER was not clearly demonstrated. In 2015, Qin et al. reported a type of POMOFs called [TBA]_3_[ε‐PMo^V^_8_Mo^VI^_4_O_36_(OH)_4_Zn_4_][BTB]_4/3_·*x*Guest (NENU‐500, BTB = benzene tribenzoate, TBA^+^ = tetrabutylammonium ion) as an ultrastable electrocatalyst for the HER.^92^ It displayed a Tafel slope of 96 mV dec^−1^ and an overpotential of 237 mV at a current density of 10 mA cm^−2^ (a metric associated with solar fuel synthesis), which was inferior to Pt/C (Tafel slope of 30 mV dec^−1^ and overpotential of 52 mV at 10 mA cm^−2^). Very recently, Dai et al. demonstrated MoS_x_ anchored on Zr‐MOF (UiO‐66‐NH_2_) prepared by a solvothermal method for the HER.^93^ The introduction of MoS_x_ nanosheets to the MOFs dramatically enhanced the HER activity due to the improved electron transport, the increased number of active sites and the favorable delivery of local protons in the Zr‐MOF structure. By optimizing the MoS_x_ amount, the MoS_x_‐MOF composite with a Mo/Zr ratio of 0.5 displayed remarkable HER activity, with a Tafel slope of 59 mV dec^−1^, which was only slightly higher than that of Pt/C (32 mV dec^−1^).^93^

**Figure 1:**
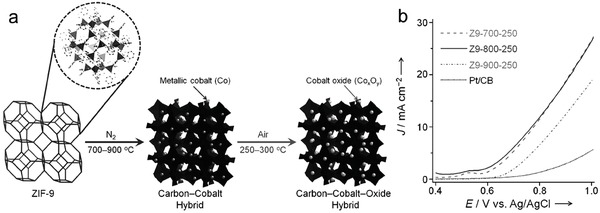
a) Schematic presentation of the formation of Co_x_O_y_‐C hybrids through the two‐step thermal treatment of ZIF‐9. b) Polarization curves of Z9‐700‐250, Z9‐800‐250, Z9‐900‐250, and Pt/CB as electrocatalysts for the OER in 0.1 M KOH. The resulting hybrids were designated as Z9‐x‐y, where x and y stand for the thermal treatment temperatures in the first and second steps, respectively. Reproduced with permission.^94^

In the study of MOFs as precursors for the preparation of electrocatalysts for water splitting reactions, Chaikittisilp and co‐workers were the first to use a Co‐based MOF (zeolitic imidazolate framework‐9, ZIF‐9) as a precursor for the preparation of a nanoporous Co_x_O_y_‐C hybrid as an electrocatalyst for the OER.^94^ The conversion of ZIF‐9 to the Co_x_O_y_‐C hybrid is shown in **Figure** **1**a. As depicted in Figure 1b, for the OER, the Z9‐700‐250 and Z9‐800‐250 electrocatalysts exhibited more negative onset potentials and higher current densities than Z9‐900‐250 and Pt/carbon black. These results indicated that the Z9‐800‐250 hybrid is a promising electrocatalyst for the OER. Very recently, Aijaz et al. reported a highly active electrocatalyst for the OER comprising core‐shell Co@Co_3_O_4_ NPs embedded in CNT‐grafted N‐doped carbon‐polyhedra, which was obtained by the pyrolysis of a Co‐based MOF in H_2_ atmosphere and a subsequent controlled oxidative calcination.^95^ This electrocatalyst displayed an overpotential of 410 mV at 10 mA cm^−2^, comparable to RuO_2_, which has been demonstrated as the benchmark electrocatalyst for the OER.

Although nanoporous carbon was successfully synthesized from MOFs in 2008^96^ and was widely used in the oxygen reduction reaction (ORR),^97^, ^98^, ^99^ the direct use of nanoporous carbon derived from MOFs in water splitting was demonstrated only recently by Xia et al.^100^ In that study, the pyrolysis synthesis of hollow nitrogen‐doped carbon nanotube frameworks (NCNTFs) derived from a Co‐based MOF (ZIF‐67) was conducted, which provided the C and N source for the growth of N‐doped CNT catalyzed by the metallic Co NPs formed in situ and served as the template for the formation of the hollow framework.^100^ The as‐prepared NCNTFs exhibited remarkable electrocatalytic activity and stability for the OER in an alkaline medium.

Because the catalytic activity of the electrocatalysts for the HER and OER is strongly related to the morphology/microstructure, the number of active sites, the BET surface area and the charge transfer capability, modifications of MOF‐based or MOF‐derived electrocatalysts with special morphologies/nanostructures, high surface areas, abundant active sites and excellent charge transfer capability has been exploited in the last five years. In the following section, recent progress in the design of MOFs as catalysts for electrocatalytic water splitting is summarized.

#### 3.1.1 Morphology Control/Nanostructuring

Among the various parameters, the morphology of the electrocatalyst, which can provide more active sites and enhance the surface adsorption capability of the reactants, plays a crucial role in the activity for water splitting.^38^, ^39^, ^101^ In general, the morphology of MOF‐based catalysts can be tuned through the preparation method. For example, a Zn‐based MOF, MOF‐5, was prepared by an ionic liquid (IL)‐based method and displayed a distinct flower‐shaped morphology with a diameter of approximately 10 µm, very different from the regular cubic structure of MOF‐5 prepared by traditional methods. The as‐prepared MOF‐5(IL) displayed superior activity for the HER compared to cubic MOF‐5 due to the enhanced oxidation desorption reaction of the hydrogen atoms.^101^

**Figure 2:**
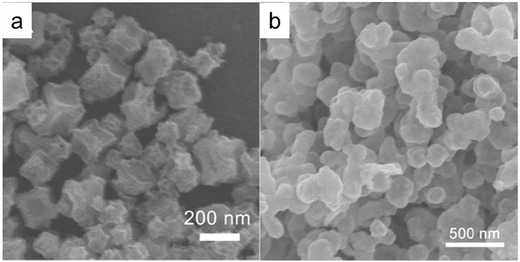
Scanning electron microscopy (SEM) images of CoP CPHs (a) and CoP NPs (b). Reproduced with permission.^102^ Copyright 2015, Royal Society of Chemistry.

In addition to the direct use of MOFs in electrocatalytic water splitting, MOFs have also been applied as precursors for the synthesis of electrocatalysts with controlled particle sizes and morphologies.^102^, ^103^, ^104^, ^105^ The morphology of the electrocatalysts derived from MOFs precursors can be tailored through the choice of the type of MOF and the temperature and atmosphere used for the subsequent calcination. For example, a CoP electrocatalyst with a concave polyhedrons (CPHs) morphology was synthesized by topological conversion using ZIF‐67 polyhedrons as the precursor.^102^ The morphology of the CoP CPHs is shown in **Figure** **2**a. CoP NPs synthesized from direct calcination of Co(NO_3_)_2_ show a different morphology (Figure 2b). For the HER, the CoP CPHs electrocatalyst showed a low overpotential of 133 mV at 10 mA cm^−2^, while the CoP NPs had an overpotential of 187 mV under the same conditions. In addition, the Tafel slopes of the CoP CPHs and CoP NPs are 51 and 63 mV dec^−1^, respectively. The electrocatalytic performance of the porous CoP CPHs is superior to most of the reported non‐noble‐metal‐based catalysts, such as CoP microspheres and FeP nanosheets, for the HER.^106^, ^107^

**Figure 3:**
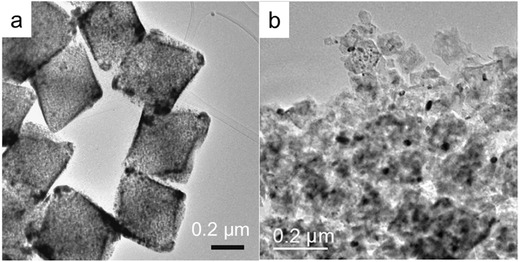
Transmission electron microscopy (TEM) images of MoC_x_ nano‐octahedrons (a) and MoC_x_ NPs (b). Reproduced with permission.^108^ Copyright 2015 Nature Publishing Group.

In addition to CoP CPHs, electrocatalysts with other controlled morphologies, such as nano‐octahedrons, spindle‐like 3D structures and porous nanocages, were also synthesized from MOFs and were investigated as electrocatalysts for the OER and HER, showing attractive activity.^108^, ^109^, ^110^ For example, Wu et al. introduced a MOF‐assisted strategy for the synthesis of MoC_x_ nano‐octahedrons as electrocatalysts for the HER.^108^ The MoC_x_ nano‐octahedrons (**Figure** **3**a) exhibited superior electrocatalytic activity and stability compared to irregular‐shaped MoC_x_ NPs with a similar composition (Figure 3b) in both acidic and basic solutions. The overpotential of MoC_x_ nano‐octahedrons was 87 and 92 mV at 1 mA cm^−2^ in acidic and basic solutions, respectively, while it was approximately 230 mV for the irregular‐shaped MoC_x_ NPs in acidic and basic solutions.

**Figure 4:**
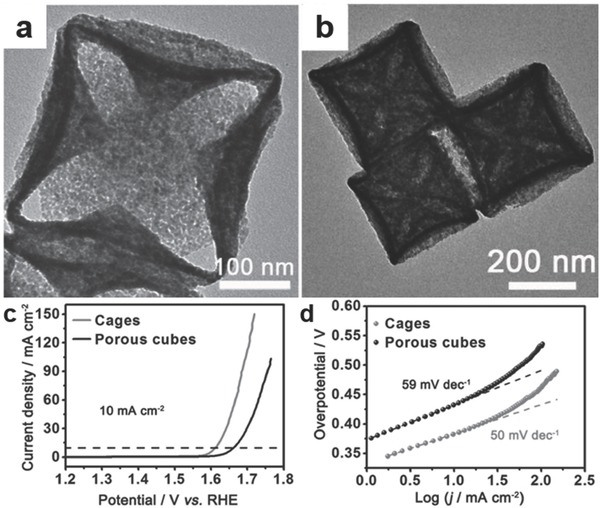
TEM images of the as‐prepared Ni‐Co oxide nanocages (a) and porous nanocubes (b); Polarization curves (c) and Tafel plots (d) of the Ni‐Co mixed oxide nanocages and porous nanocubes for the OER. Reproduced with permission.^110^

In another study, MOF‐derived Ni‐Co‐based metal oxides with nanocage morphology were compared with a catalyst with a nanocube morphology and the same composition and comparable surface area (≈31 m^2^ g^−1^) for water splitting.^110^ Ni‐Co Prussian‐blue‐analog (PBA) nanocages were derived from the Ni‐Co‐PBA nanocubes as precursors and were converted to Ni‐Co mixed oxides by calcination in air. As shown in **Figure** **4**a,b, the Ni‐Co mixed oxides inherited the nanocage morphology of the Ni‐Co PBA particles but had rougher surfaces than the pristine Ni‐Co mixed oxide nanocubes. As shown in Figure 4c,d, the Ni‐Co mixed oxide nanocages exhibited lower onset potential, lower Tafel slope and higher current density than the nanocubes for the OER. The enhanced OER activity of the Ni‐Co mixed oxide nanocages was assigned to the hollow and porous nanocage morphology.

#### 3.1.2 Constructing Hybrids/Composites

Because of the large variation of organic moieties in MOFs, highly nanoporous carbon may be formed by thermal carbonization of MOFs in inert atmospheres.^111^, ^112^ Such MOF‐derived nanoporous carbon materials usually exhibit exceptionally high surface areas, which can form strong coupling with metal oxides/metal to enhance the electrocatalytic activity and stability for water splitting.^67^, ^70^, ^113^, ^114^, ^115^, ^116^, ^117^ Thus, many investigations have attempted the synthesis of carbon‐metal/metal oxide composites from MOFs as electrocatalysts for the HER and OER. For example, hybrid porous nanowire arrays (NAs) composed of strongly interacting Co_3_O_4_ and carbon (Co_3_O_4_C‐NA) with a high specific surface area of 251 m^2^ g^−1^ and a large carbon content of 52.1 wt.% were successfully prepared by a facile carbonization of MOF grown on Cu foil,^113^ and displayed superior performance as the working electrode for OER without additional substrates or binders. A low onset potential of 1.47 V vs. RHE and a stable current density of 10 mA cm^−2^ at 1.52 V vs. RHE in 0.1 M KOH for 30 h were achieved. Co_3_O_4_ C‐NA also showed a much lower Tafel slope of 61 mV dec^−1^ than IrO_2_/C (87 mV dec^−1^) in 1 M KOH. For comparison, the carbon‐free counterpart (Co_3_O_4_‐NA), prepared by the calcination of Co_3_O_4_C‐NA in air, which exhibited a similar porous NA structure and a cubic spinel phase, displayed a higher onset potential of 1.50 V vs. RHE, a larger operating potential of 1.64 V vs. RHE at 10 mA cm^−2^ and a much higher Tafel slope of 123 mV dec^−1^. Co_3_O_4_C‐NA delivered superior OER activity and stability compared with the state‐of‐the‐art noble‐metal electrocatalyst.^113^

**Figure 5:**
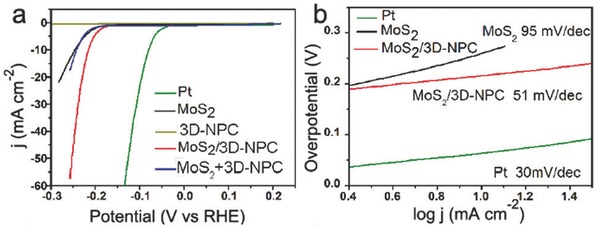
a) Polarization curves and b) Tafel plots of the MoS_2_/3D‐NPC composite, 3D‐NPC, MoS_2_ NPs, mixed MoS_2_+3D‐NPC and Pt. Reproduced with permission.^118^ Copyright 2015, Royal Society of Chemistry.

The strong coupling was utilized for the development of a MoS_2_‐based composite (MoS_2_/3D‐NPC) as the electrocatalyst for the HER, in which the MoS_2_ nanosheets grew in situ in the nanopores of 3D nanoporous carbon (3D‐NPC) derived from MOF and showed much better performance than the respective components (MoS_2_ NPs, 3D‐NPC) of the catalyst, as well as the physically mixed MoS_2_ NPs and NPC composite (MoS_2_+3D‐NPC).^118^ The MoS_2_/3D‐NPC composite displayed a small overpotential of 210 mV at 10 mA cm^−2^ for the HER, better than those of 3D‐NPC (>300 mV), MoS_2_ NPs (≈250 mV) and MoS_2_ +3D‐NPC (≈250 mV) as shown in **Figure** **5**a. The Tafel slope of the MoS_2_/3D‐NPC composite reached 51 mV dec^−1^, in contrast to 95 mV dec^−1^ for the MoS_2_ NPs as shown in Figure 5b. The high electrocatalytic activity of the MoS_2_/3D‐NPC electrocatalyst was attributed to the efficient charge transfer in the HER due to the more exposed active sites and robust interaction between the MoS_2_ and the MOF‐derived conductive carbon when the MoS_2_ nanosheets were grown in the pores of the 3D‐NPC.

**Figure 6:**
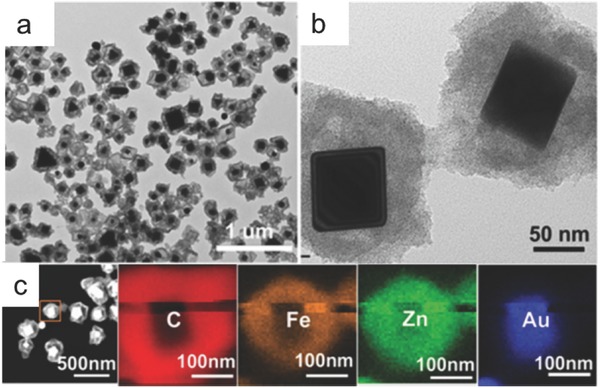
TEM images (a, b) and energy‐dispersive X‐ray spectroscopy (EDX) mapping (c) of Au@Zn‐Fe‐C hybrids. Reproduced with permission.^119^ Copyright 2016 American Chemical Society.

In another study, Lu et al. reported the development of a core (Au NP, ≈50–100 nm)‐shell (Zn‐Fe‐C, ≈30–60 nm) composite (**Figure** **6**a–c) prepared by direct pyrolysis of a Zn‐Fe‐MOF shell coated on an Au NP in an inert atmosphere.^119^ The core‐shell catalyst (Au@Zn‐Fe‐C) displayed a low onset potential of ‐0.08 V vs. RHE in 0.5 M H_2_SO_4_, which was much more positive than those of Au (‐0.225 V) and Zn‐Fe‐C (‐0.292 V) but slightly more negative than that of commercial Pt/C (‐0.006 V). The Tafel slope of Au@Zn‐Fe‐C was 130 mV dec^−1^, which was lower than the slopes of Au (167 mV dec^−1^) and Zn‐Fe‐C (271 mV dec^−1^). The onset overpotential and lower Tafel slope contributed to the more favorable HER kinetics of Au@Zn‐Fe‐C, demonstrating a synergistic effect between the Au NP core and Zn‐Fe‐C shell.

#### 3.1.3 Functional Modification

The charge transfer capability of the electrocatalyst plays a critical role in determining the activity for OER and HER. For MOF‐based catalysts, the addition of functional ions and/or ligands can enhance the charge transfer capability. For example, Wang et al. developed a new electrocatalyst by assembling Co ions and benzimidazolate ligands into a MOF (Co‐ZIF‐9) for the OER with high proton transfer capability.^120^ Co‐ZIF‐9 was effective for the electrocatalytic OER, and its turnover frequency (TOF) reached ≈1.76 × 10^−3^ s^−1^, which is similar to the active Co‐based electrocatalyst reported in the literature.^121^ Wang et al.'s work made an important step in water splitting chemistry by integrating the redox‐active metal centers and organic motifs into a MOF structure.

In addition to the design of MOFs, some carbon‐based functional additives were incorporated into MOF‐based catalysts to enhance the charge transfer capability.^122^, ^123^ For example, GO was used to modify the MOFs to enhance the charge transfer capability and improve the electrocatalytic activity for water splitting.^123^ The GO‐incorporated Cu‐MOF composite displayed good performance as a bifunctional catalyst for the HER and OER in 0.5 M H_2_SO_4_.^123^ The (GO 8 wt.%) Cu‐MOF exhibited the highest activity for the HER. The onset overpotential for the HER was 202 mV for Cu‐MOF and decreased from 123 to 87 mV as the GO amount increased from 2 to 8 wt.%. For the OER, the Tafel slope was 65 mV dec^−1^ for (GO 8 wt.%) Cu‐MOF and 89, 81 and 61 mV dec^−1^ for Cu‐MOF, (GO 2 wt.%) Cu‐MOF and Pt/C, respectively. The improved activity of the GO‐MOF composite for water splitting in acidic solution was attributed to the enhanced charge transfer capability and synergistic effects of GO and MOF.

Functional carbon‐based materials were also added to MOF‐based materials in the precursor stage for the preparation of highly active MOF‐derived electrocatalysts.^73^, ^124^ For example, Tang et al. synthesized an active electrocatalyst derived from a POMOFs/GO composite for the HER.^73^ The introduction of GO to POMOFs improved the conductivity and the HER activity of MoO_2_@PC‐RGO and functioned as a support for the formation of a closely connected network. This hybrid presented superior activity for the HER in acidic media due to the synergistic effects among the MoO_2_ NPs, PC and RGO substrates.^73^ MoO_2_@PC‐RGO initiated H_2_ evolution near its thermodynamic overpotential (0 mV), which was similar to that of the Pt/C catalyst, while MoO_2_@PC had an onset overpotential of 66 mV. The overpotentials at 10 mA cm^−2^ were 38 mV for Pt/C, 64 mV for MoO_2_@PC‐RGO and 136 mV for MoO_2_@PC. Tafel slopes of 30, 41 and 60 mV dec^−1^ were obtained for Pt/C, MoO_2_@PC‐RGO and MoO_2_@PC in 0.5 M H_2_SO_4_.

**Figure 7:**
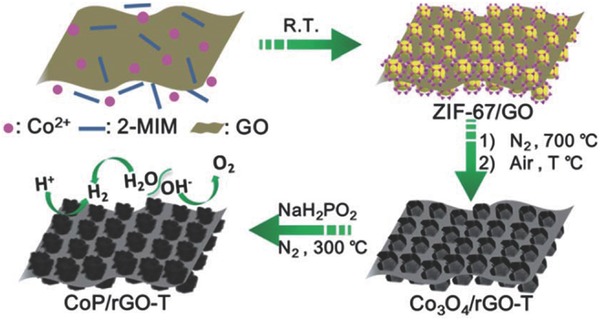
Illustration of the fabrication procedure for the CoP/rGO nanocomposite. Reproduced with permission.^124^ Copyright 2016, Royal Society of Chemistry.

In another work, rGO was incorporated with CoP to enhance the charge transfer capability of CoP for the HER and OER in alkaline media.^124^ This layered composite was prepared by pyrolysis and phosphating processes with rationally designed sandwich‐type ZIF‐67 MOF/GO as a template and precursor, as shown in **Figure** **7**. The MOF‐derived porous CoP nanostructure guaranteed a large quantity of exposed active sites, and the close contact between CoP and rGO contributed to a continuous conductive network, which was beneficial for the electron transfer process. For the HER, CoP/rGO‐400 was more active than rGO and CoP due to the synergistic effect between the CoP and rGO in the composite. In 1 M KOH, a low Tafel slope of 38 mV dec^−1^ was achieved with the CoP/rGO‐400 electrocatalyst, comparable to that of Pt/C (36 mV dec^−1^) and much lower than CoP (60 mV dec^−1^). For the OER in the same alkaline solutions, CoP/rGO‐400 displayed a Tafel slope of 66 mV dec^−1^, which was superior to CoP, rGO and even the state‐of‐the‐art IrO_2_. These contributions shed light on the rational design of a series of RGO‐incorporated MOF‐based electrocatalysts for the OER and HER.

#### 3.1.4 Intrinsically Conductive MOFs

MOF is typically not conductive and may not be a great choice for electrocatalysis‐based applications. As such, one of the most challenging and rewarding endeavors in this field is to synthesize porous MOFs with good charge mobility and conductivity. Recently, the development of new type MOFs with enhanced intrinsic conductivity has attracted increasing attention.^125^, ^126^, ^127^ In 2009, Takaishi et al. reported one of the first conductive MOFs, Cu[Cu(pdt)_2_] (pdt = 2,3‐pyrazinedithiolate), and its electrical conductivity was 6 × 10^−4^ S cm^−1^ at 300 K.^128^ However, this kind of MOF collapsed upon desolvation. Thus, it is critical to develop alternative MOFs with high conductivity and structural stability for the potential electrocatalysis application.

Dincă and co‐workers have made great contributions to the development of conductive MOFs for the potential use in electrocatalysis‐based applications.^129^ For example, Sun et al. treated Mn^2+^ with 2,5‐disulfhydrylbenzene‐1,4‐dicarboxylic acid (H_4_DSBDC) and obtained isolated Mn_2_(DSBDC).^130^ This MOF was a thiolated analogue of Mn_2_(DOBDC) MOF derived from 2,5‐dihydroxybenzene‐1,4‐dicarboxylic acid (H_4_DOBDC). The porous Mn_2_(DSBDC) with one‐dimensional (1D) (–Mn–S–)_∞_ chains showed a high surface area (978 m^2^  g^−1^) and high charge mobility (0.01 cm^2^  V^−1^  s^−1^) similar to those of the most common organic semiconductors. Although Mn_2_(DSBDC) displayed a relatively high charge mobility, its conductivity was rather low (3.9 × 10^−13^ S  cm^−1^ at 297 K). The replacement of d^5^ Mn^II^ by d^6^ Fe^II^ centers introduces high energy, loosely bound minority‐spin carriers and then enhances the conductivity.^131^ They found that the bulk electrical conductivity values of both Fe_2_(DSBDC) and Fe_2_(DOBDC) were ≈6 orders of magnitude higher than those of the Mn^2+^ analogues, Mn_2_(DOBDC) and Mn_2_(DSBDC), which was attributed to the loosely bound Fe^2+^β‐spin electron. This study can provide important insights for the rational design of conductive MOFs, highlighting in particular the advantages of iron for synthesizing conductive MOF‐based materials.

Besides Fe, Cd was also demonstrated to be a promising candidate to construct conductive MOFs. Park et al. found that isostructural MOFs M_2_(TTFTB) (M = Mn, Co, Zn and Cd; H_4_TTFTB = tetrathiafulvalene tetrabenzoate) exhibited a striking correlation between their single‐crystal conductivities and the shortest S···S interaction defined by neighboring tetrathiafulvalene (TTF) cores, which inversely correlated with the ionic radius of the metal ions.^132^ The Cd analogue, with the largest cation and shortest S···S contact, showed the highest electrical conductivity (2.86 × 10^−4^ S cm^−1^) that was 72 times higher than that of Zn_2_(TTFTB) (3.95 × 10^−6^ S cm^−1^). Mn_2_(TTFTB) and Co_2_(TTFTB), which display intermediate S···S distances between those observed in the Zn and Cd analogues, also showed intermediate conductivity values of 8.64 × 10^−5^ and 1.49 × 10^−5^ S cm^−1^, respectively, both tracking inversely with increasing S···S distance.

Highly conductive two‐dimensional (2D) MOFs made from nitrogen‐based ligands were reported in 2014 by Sheberla et al.^133^ They found that the reaction of NiCl_2_ with hexaaminotriphenylene (H_6_HATP) in aqueous NH_3_ solution led to the isolation of a new MOF, Ni_3_(HITP)_2_ (HITP = 2,3,6,7,10,11‐hexaiminotriphenylene). Ni_3_(HITP)_2_ films grown on a quartz substrate displayed a conductivity of 40 S cm^−1^ at room temperature, while pellets of the same material showed a bulk conductivity of 2 S cm^−1^. The isostructural material made from Cu^II^, Cu_3_(HITP)_2_, also displayed a high bulk conductivity of 0.2 S cm^−1^ at room temperature.

The large bulk conductivity of HITP‐based materials enabled the application in chemiresistive sensing for ammonia vapor or volatile organic compounds (VOCs).^134^, ^135^ Cu_3_(HITP)_2_ functioned as reversible chemiresistive sensors, which was capable of detecting sub‐ppm levels of ammonia vapor.^134^ It was found that the chemiresistive response can be altered by the choice of metal node and Ni_3_(HITP)_2_ was unresponsive to ammonia, suggesting that the copper sites are critical for ammonia sensing. These studies highlight the utility of 2D conductive MOFs in the production of tunable functional materials, such as chemical sensors.

Very recently, Miner et al. investigated an intrinsically conductive Ni_3_(HITP)_2_ MOF as an active, well‐defined and tunable electrocatalyst toward the ORR in alkaline solutions.^136^ Ni_3_(HITP)_2_ exhibited ORR activity competitive with the most active Pt‐free electrocatalysts due to the combination of high crystallinity of MOFs, the physical durability and electrical conductivity of graphitic materials, and the diverse, well‐controlled synthetic accessibility of molecular species. In addition, the Ni_3_(HITP)_2_ MOF with high electrical conductivity is directly used as the electrode material in electrochemical double layer capacitors (EDLCs) without conductive additives or other binders.^137^ The performance of this MOF exceeded that of most carbon‐based materials with capacity retention greater than 90% over 10000 cycles, suggesting conductive MOFs as a new generation of active materials for supercapacitors. These studies highlight the direct use of conductive MOFs in fuel cells and supercapacitors, which can shed light to their future application in the electrocatalytic water splitting.

#### 3.1.5 A Brief Summary

The catalytic activities of some typical MOFs and MOF‐derived electrocatalysts for the HER and/or OER are summarized in **Table** **1**. Taking advantage of the porous structures of MOF precursors, the MOF‐derived materials had various morphological features, such as polyhedrons, nanocubes, nanocages, nano‐octahedrons, nanosheets and nanowires, and outperformed their NP counterparts of similar compositions for catalyzing the water splitting reactions. Remarkably, in the HER under acidic conditions, porous CoP with a CPHs structure prepared from Co‐MOFs exhibited a >50 mV decrease in the overpotential required to reach 10 mA cm^−2^ compared to CoP NPs.^102^ As MOFs typically have abundant carbon and nitrogen sources, the carbonation of MOFs can lead to the formation of catalytically active metal‐based material composites with carbon materials having finely tuned porosity, graphitic degree and nitrogen content (if any). These hybrids have demonstrated good HER/OER performance, as shown in Table 1. Particularly, a strongly interacting Co_3_O_4_ and carbon hybrid grown on Cu foil directly catalyzed the OER with high efficiency, reaching 10 mA cm^−2^ at an overpotential as low as 290 mV in 0.1 M KOH.^113^ In addition to applying MOFs alone to synthesize electrocatalytic materials, GO and rGO were functionalized with MOFs to improve the charge transfer of the catalysts, as well as the catalytic performance. Despite the effectiveness of MOF‐derived materials in the HER and/or OER, most can only promote the HER in acidic media (while industrial water splitting favors an alkaline solution) and few can catalyze the overall water splitting process (both HER and OER) in the same electrolyte. Therefore, more efforts should be made to design bifunctional electrocatalysts from MOFs with the combined knowledge of MOF structures and the mechanism of the water splitting process. More importantly, current MOF‐derived catalysts can only sustain constant hydrogen or oxygen production within limited timescales, ranging from several hours to a few days. Robust MOF‐derived materials that can operate on a longer timescale are thus needed to go a step further toward the real application of water splitting.

**Table 1:** Electrocatalytic activity of some typical MOF‐based and MOF‐derived electrocatalysts for the HER and OER

No.	Electrocatalyst	Target reaction	Medium	Overpotential at 10 mA cm^−2^ (mV)	Tafel slope (mV dec^−1^)	Durability (h)	Ref.
1	(GO 8 wt%) Cu‐MOF composite	OER	0.5 M H_2_SO_4_	110 at 2 mA cm^−2^	65	/	123
		HER		209 at 30 mA cm^−2^	84	/	
2	N‐doped carbon nanotube frameworks (NCNTFs)	OER	1 M KOH	370	93	≈3	100
3	Zn_0.30_Co_2.70_S_4_ polyhedra	HER	0.5 M H_2_SO_4_	80	47.5	60	66
4	Co_3_S_4_ polyhedra			380	85.3	10	
5	CoP CPHs	HER	0.5 M H_2_SO_4_	133	51	12	102
6	MoC_x_ nano‐octahedrons	HER	0.5 M H_2_SO_4_	142	53	11	108
			1 M KOH	151	59	11	
7	Ni‐Co mixed oxide cages	OER	1 M KOH	380	50	10	110
8	Ni‐Co mixed oxide cubes			430	59	10	
9	CoNi hydroxide ultrathin nanosheets	OER	1 M KOH	324	33	≈3	104
10	Spindle‐like Co/Fe metal oxides in N‐doped porous carbon	OER	0.1 M KOH	390	72.9	/	109
11	CoP_x_ NPs embedded in N‐doped carbon matrices	OER	1 M KOH	319	52	24	67
		HER		154	51	24	
12	Co_3_O_4_C‐NA	OER	0.1 M KOH	290	70	30	113
13	MoS_2_/3D‐NPC	HER	0.5 M H_2_SO_4_	210	51	≈3	118
14	Au@Zn−Fe−C	HER	0.5 M H_2_SO_4_	123	130	12	119
15	Atomically isolated nickel species anchored on graphitized carbon	HER	0.5 M H_2_SO_4_	34	41	25	117
16	Co NPs embedded in porous N‐rich carbon	OER	1 M KOH	370	76	10	115
		HER		298	131	10	
17	Co@Co_3_O_4_ encapsulated in CNT‐grafted N‐doped carbon polyhedra	OER	0.1 M KOH	410	54.3	45	95
18	Layered CoP/rGO composite	HER	0.5 M H_2_SO_4_	105	50	22	124
			1 M KOH	150	38	22	
		OER	1 M KOH	340	66	22	

### 3.2 Photocatalytic Water Splitting

**Figure 8:**
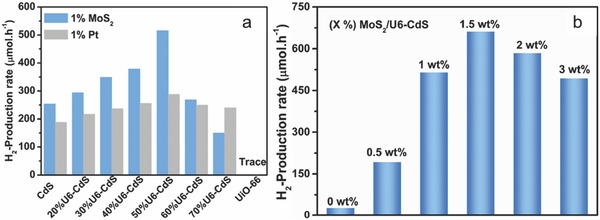
a) The H_2_ production rate over CdS, UiO‐66 and UiO‐66‐CdS composites loaded with 1 wt.% MoS_2_ or Pt. b) The H_2_ production rate over UiO‐66‐CdS composite (1:1, w/w) loaded with different amounts of MoS_2_. Reproduced with permission.^143^ Copyright 2015, Elsevier.

Some MOFs have shown favorable photocatalytic activity for water splitting, dye degradation, CO_2_ reduction and Cr(VI) reduction.^138^, ^139^, ^140^, ^141^ For photocatalytic water splitting, the direct use of MOFs as photocatalysts for the HER was first reported in 2010 by Silva et al.^138^ They demonstrated two types of Zr‐based MOFs formed by terephthalate (UiO‐66) and 2‐aminoterephthalate ligands (UiO‐66‐NH_2_) as stable photocatalysts for the HER. However, the H_2_ generation rates of both photocatalysts were low (248 and 372 µmol h^−1^ g^−1^ for UiO‐66 and UiO‐66‐NH_2_, respectively). Thus, it is critical to incorporate a Pt co‐catalyst and to optimize the Pt amount to enhance the activity of MOF‐based photocatalysts.^142^ The optimum Pt amount (1.5 wt.%) achieved a high H_2_ evolution rate of 500 µmol h^−1^ g^−1^ with a Pt/Ti‐MOF‐NH_2_ photocatalyst.^142^ Recently, Shen et al. used an active MoS_2_ to replace expensive Pt in the UiO‐66‐CdS system, and superior photocatalytic activity was achieved with the MoS_2_/UiO‐66‐CdS photocatalyst.^143^ As shown in **Figure** **8**a, the incorporation of CdS and UiO‐66 enhanced the photocatalytic activity of both parts of the reaction, and the optimum HER activity was obtained at a UiO‐66 to CdS weight ratio of 1:1. A high H_2_ evolution rate of 25,770 µmol h^−1^ g^−1^ was obtained in the presence of 1 wt.% MoS_2_, approximately 2‐fold higher than that of 1 wt.% Pt/UiO‐66‐CdS. As shown in Figure 8b, the H_2_ evolution rate of MoS_2_/UiO66‐CdS reached a maximum when the MoS_2_ amount in the composite was 1.5 wt.% (32,500 µmol h^−1^ g^−1^). Shen et al.'s work demonstrated the advantage of using MoS_2_ as a highly active co‐catalyst to replace Pt in MOF‐based photocatalysts to enhance the HER activity.

Lin and co‐workers were the first to use an Fe‐based MOF (MIL‐101) coated with amorphous TiO_2_ as a template to prepare Fe_2_O_3_@TiO_2_ composite as a photocatalyst for the HER.^144^ This MOF‐derived Fe_2_O_3_@TiO_2_ composite exhibited superior HER activity to either Fe_2_O_3_ or TiO_2_ alone and a mixture of the two. However, this composite had a low H_2_ evolution rate of ≈600 µmol h^−1^ g^−1^ after depositing Pt NPs as co‐catalysts. More recently, Pham et al. reported a hollow Fe_2_O_3_‐TiO_2_‐PtO_x_ photocatalyst for the HER that was synthesized from MIL‐88B as a template.^145^ The hollow Fe_2_O_3_‐TiO_2_‐PtO_x_ nanocomposite displayed an enhanced hydrogen evolution rate of 1100 µmol h^−1^ g^−1^ by separating the two co‐catalysts (Fe_2_O_3_ and PtO_x_) on two surface sides of TiO_2_. In 2015, Bala et al. reported a MOF‐derived Co_3_O_4_/TiO_2_ photocatalyst with rich p‐n heterojunctions for the HER.^88^ The Co_3_O_4_/TiO_2_ composite with the optimum Co loading amount of 2 wt.% exhibited the maximum photocatalytic activity, with a hydrogen evolution rate of 7000 µmol g^−1^ h^−1^, which was almost 7 times greater than that of the conventional Co_3_O_4_/TiO_2_ nanocomposite.^88^

As demonstrated previously, the catalytic activity of photocatalysts for the HER and OER is strongly related to the band gap energy, the visible light absorption capability, the composition and quantity of active sites/co‐catalysts and the charge transfer capability. In the following section, we summarize several important examples of MOFs as photocatalysts for water splitting under solar irradiation, including band gap engineering to improve the visible light response, active site/co‐catalyst selection, incorporation and optimization to enhance the activity and structural evolution, functional coupling and the creation of heterojunctions to enhance the charge transfer capability.

#### 3.2.1 Engineering the Band Gap and/or Enhancing the Visible Light Adsorption Capability

Tremendous effort has been made to develop photocatalysts with high activity under visible light irradiation, and it is primarily important to tailor the band structure.^34^, ^35^, ^36^, ^37^ A hybrid organic/inorganic compound with a MOF structure, Cu_3_PO_4_(C_2_N_3_H_2_)_2_OH, which was synthesized by hydrothermal route in the presence of 1,2,4‐triazole as a structure directing agent, was investigated as a photocatalyst for the OER under visible light.^146^ This Cu‐based MOF had a band gap of 2.58 eV, which ensured visible‐light‐driven photocatalytic activity. When Cu_3_PO_4_(C_2_N_3_H_2_)_2_OH was used as the photocatalyst, the OER was successful under visible light irradiation, with an O_2_ evolution rate of ≈1875 µmol h^−1^ g^−1^. In principle, MOFs with open metal coordination sites might be able to activate small molecules, such as water, triggering chemical reactions under mild conditions. Therefore, the water oxidation reaction based on this type of porous material should be explored.

**Figure 9:**
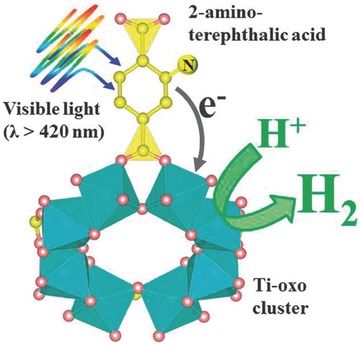
Schematic illustration of photocatalytic hydrogen production reaction over Ti‐MOF‐NH_2_ under visible light irradiation (λ > 420 nm). Reproduced with permission.^142^ Copyright 2013, Royal Society of Chemistry.

In an attempt to enhance the activity of MOF‐based photocatalysts for the HER under visible light irradiation, Toyao et al. reported a Ti‐MOF‐NH_2_ photocatalyst using 2‐amino‐benzenedicarboxylic acid (H_2_BDC‐NH_2_) as an organic linker (**Figure** **9**) for the photocatalytic HER under visible light irradiation.^142^, ^147^ Ti‐MOF showed an absorption edge of 350 nm, while the edge of Ti‐MOF‐NH_2_ reached ≈500 nm. In the photocatalytic process, the organic linker absorbed visible light, and the electrons transferred from its excited state to the CB of the photoactive titanium‐oxo cluster, as shown in Figure 9. Pt/Ti‐MOF‐NH_2_ exhibited efficient photocatalytic activity for the HER under visible light irradiation, and a H_2_ evolution rate of ≈367 µmol h^−1^ g^−1^ was obtained. In contrast, Pt/Ti‐MOF displayed no photocatalytic activity for the HER. These results suggested that the band gap/visible light response played a critical role in the photocatalytic water splitting. However, the structural stability of Pt/Ti‐MOF‐NH_2_ should be carefully addressed in the future.

Although MOF‐based photocatalysts with organic linkers were successfully developed for water splitting under visible light, the longest wavelength available was only 500 nm due to the limited visible light response and low oxidation power of the BDC‐NH_2_ organic linker.^142^, ^147^ Thus, it is critical to extend the wavelength range by using other organic linkers that have wide visible light absorption and high oxidation power. Bis(4′‐(4‐carboxyphenyl)‐terpyridine) Ru(II) (Ru(tpy)_2_) was found to have wider absorption in the visible light region and a lower highest occupied molecular orbital (HOMO) level than BDC‐NH_2_. A Ru complex‐modified Ti‐based MOF (Ti‐MOF‐Ru(tpy)_2_) was synthesized and exhibited high photocatalytic activity for the HER under visible light, with a wavelength up to 620 nm.^148^

In addition to the reduction in the band gap of MOF, the visible light response can be increased by developing a dye‐sensitized system.^44^, ^149^ Since both MOFs and dyes contain a benzene ring, the strong π–π stacking and van der Waals interactions between MOFs and dyes could enhance the electron transfer in a dye‐sensitized system.^150^ For example, a rhodamine B (RhB)‐sensitized MOF was developed for the HER.^149^ RhB‐sensitized UiO‐66 exhibited high activity for HER under visible light irradiation with a H_2_ evolution rate of 116 µmol h^−1^ g^−1^, which was 30 times greater than that of Pt@UiO‐66.^149^ However, the poor stability of RhB restricted its application as an effective photosensitizer.^151^ Recently, Erythrosin B (ErB) dye, with superior stability to RhB, was selected to sensitize UiO‐66 MOF for photocatalytic H_2_ production.^44^ With the optimized amount of ErB, Pt‐UiO‐66‐30 (ErB/photocatalyst weight ratio of 3) displayed the highest H_2_ evolution rate of 460 µmol h^−1^ g^−1^, while Pt‐UiO‐66 without sensitization showed no HER activity. In addition, the photocatalytic activity of ErB‐sensitized UiO‐66 was higher than those of ErB‐sensitized P25‐TiO_2_ and SiO_2_ for the HER, demonstrating the advantage of MOFs as photocatalysts for water splitting. The enhancement in photocatalytic H_2_ production was attributed to the efficient transfer of the charge carriers from the photoexcited ErB to the MOFs. These results suggested that the dye‐sensitized MOFs showed great potential for the photocatalytic HER.

In addition to the dye‐sensitized system based on MOF‐based photocatalysts, MOF itself can be used as a photosensitizer for PEC water splitting.^152^ MIL‐125 (a Ti‐based MOF) has a more negative lowest unoccupied molecular orbital (LUMO) level than the CB edge of TiO_2_, making electron transfer from MIL‐125 to TiO_2_ feasible.^153^ The performance of TiO_2_ for PEC water oxidation was significantly enhanced by the addition of a MOF‐based photosensitizer under visible light irradiation.^152^ The photocurrent of a TiO_2_ nanowire in PEC water splitting was improved by 100% from 10 to 20 µA cm^−2^ at 0.75 V vs. RHE under visible light irradiation after the sensitization of aminated Ti‐based MOFs on TiO_2_. Considering the diversity of MOFs, a large number of MOFs are expected to function as photosensitizers for solar‐driven energy conversion. In another study, functionalized MOF‐253‐Pt was prepared by immobilizing a Pt complex in microporous MOF‐253 using a post‐synthesis method. MOF‐253‐Pt acted as both a photosensitizer and a photocatalyst for visible‐light‐driven HER.^154^ The photocatalytic activity of MOF‐253‐Pt was ≈5 times greater than that of the Pt complex, while MOF‐253 displayed almost no activity for the HER. Further studies should be conducted to design a robust and highly active MOF bifunctional photocatalyst/photosensitizer for visible‐light‐driven HER.

#### 3.2.2 Active Site/Co‐Catalyst Selection, Incorporation and Optimization

Numerous efforts have been made for the integration of different molecular functional components and MOFs to enhance water splitting activity.^83^, ^84^, ^85^, ^86^, ^87^ The combination of MOFs with functional inorganic materials, such as metal NPs and metal oxides, has been reported as a new strategy and has received increased attention.^87^, ^155^, ^156^ Some researchers incorporated Pt NPs into MOFs to enhance the water splitting activity.^87^, ^155^, ^156^ The incorporation of Pt NPs into NH_2_‐MIL‐101(Cr) was effective for visible‐light‐driven H_2_ evolution with high activity.^155^ Pt NPs with an average size of ≈3.75 nm were highly dispersed on NH_2_‐MIL‐101(Cr) with a particle size of ≈50 nm. When the Pt amount was increased from 0.5 to 1.5 wt.%, the photocatalytic activity of Pt/NH_2_‐MIL‐101(Cr) was improved due to the increased active sites, and the optimum Pt loading for H_2_ evolution was 1.5 wt.%. A further increase in the Pt amount to 3.0 wt.% decreased the activity due to the agglomeration of Pt NPs. Similarly, Pt NPs with a particle size of 1 nm were embedded into layers of a titanium picolinate framework (TiPF) by photodeposition.^156^ The obtained Pt‐loaded TiPF could be used as a photocatalyst for the HER, with a hydrogen evolution rate of 1593 µmol h^−1^ g^−1^. Furthermore, TiPF, without Pt NPs, exhibited much lower activity for the HER, with an evolution rate of 228 µmol h^−1^ g^−1^, suggesting that Pt active sites played a critical role as an electron trap to improve the charge separation efficiency and increase the HER activity. However, the stability of the TiPF support must be improved in future research.

Recently, earth‐abundant Co and Ni‐based catalysts have been used to replace the precious Pt metal in water splitting.^157^, ^158^ Compared to Pt, the control and reduction of the particle size of the oxides are more difficult. Han et al. utilized an MIL‐101 MOF to immobilize Co_3_O_4_ NPs for the OER.^157^ With cobalt loading in the range of 1.4–4.9 wt.%, ultra‐small Co_3_O_4_ NPs (2–3 nm) were successfully immobilized and well dispersed in the cages of MIL‐101. This photocatalyst displayed enhanced photocatalytic activity for the OER, with a high TOF of 0.012 s^−1^ per Co atom, which was more than 9 times higher than that of the unsupported Co_3_O_4_ NPs. Furthermore, MIL‐101 also promoted the charge transfer process in the OER.

**Figure 10:**
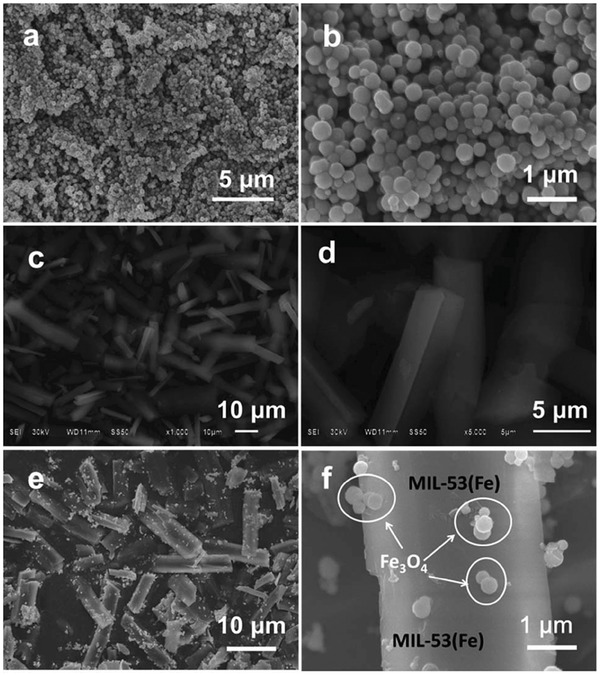
SEM images of Fe_3_O_4_ (a and b), MIL–53(Fe) (c and d) and MHMCs (e and f). Reproduced with permission.^161^ Copyright 2015, Royal Society of Chemistry.

Due to their large BET surface areas, most available MOF and NPs‐modified MOF photocatalysts are highly dispersed in water, making them difficult to recycle. It is therefore critical to develop MOF‐based photocatalysts that can be easily collected for reuse. One solution is to incorporate magnetic materials into MOFs.^159^, ^160^, ^161^ For example, a photocatalyst composed of MIL‐53(Fe) microrods with magnetic and active Fe_3_O_4_ nanosphere decoration on the surface (MIL‐53(Fe) hybrid magnetic composites, MHMCs) was reported.^161^ As shown in **Figure** **10**, the MHMCs were composed of well‐dispersed Fe_3_O_4_ nanospheres with a particle size of ≈200 nm attached to the surface of MIL‐53(Fe) microrods, creating the heterostructures. Used as a photoanode, the MHMCs delivered a remarkable photocurrent under visible light irradiation. After the OER, the used MHMCs were easily collected through magnetic separation and were recycled for further use. This structural design approach can provide an ideal platform for the development of MOF‐based materials with high activity and magnetic recyclability for energy conversion and environmental remediation.

#### 3.2.3 Structural Evolution, Coupling and Creation of Heterojunctions

The charge transfer/separation efficiency is vital to the achievement of high photocatalytic activity. Several strategies have been employed to enhance the charge transfer/separation efficiency, such as developing new MOF photocatalysts through structural evolution and coupling with carbon‐based materials to form hybrid photocatalysts.^162^, ^163^, ^164^, ^165^ For example, a highly active Bi‐based MOF photocatalyst composed of Bi^3+^ and H_2_mna (2‐mercaptonicotinic acid) was developed (denoted as Bi‐mna) for water splitting under visible light.^162^ According to the experimental and theoretical results, a ligand‐to‐ligand charge transfer (LLCT) process was responsible for the high photocatalytic activity, which resulted in a longer lifetime of the photogenerated charges, suppressed electron‐hole recombination and increased the photocatalytic activity. These results suggested that Bi‐based MOFs are promising candidates for efficient water splitting.

**Figure 11:**
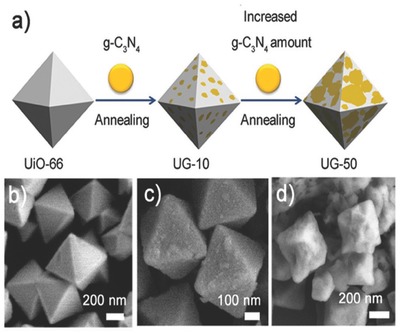
a) Schematic illustration of coating g‐C_3_N_4_ on UiO‐66 octahedrons through annealing. SEM images of b) UiO‐66, c) UG‐10 and d) UG‐50. Reproduced with permission.^163^

In addition to the design of new photocatalysts, functional additives have been used to enhance the charge transfer capability of MOF‐based photocatalysts.^163^ A new hybrid photocatalyst created by coupling UiO‐66 octahedrons and g‐C_3_N_4_ (referred to as UG‐x, where x is the weight of g‐C_3_N_4_ in the hybrids) was demonstrated.^163^ Although both g‐C_3_N_4_ and UiO‐66 were capable of the HER, the use of each material alone displayed limited photocatalytic activity due to the high charge recombination rate and poor sunlight absorption capability. **Figure** **11**a shows a schematic illustration of the coating of g‐C_3_N_4_ on UiO‐66 octahedrons by annealing. UiO‐66 had very smooth and clean surfaces as shown in Figure 11b. After annealing, g‐C_3_N_4_ nanosheets were coated on the surface of UiO‐66, making the surface uneven (Figure 11c). Increased g‐C_3_N_4_ resulted in heavy coverage of g‐C_3_N_4_ on UiO‐66 (Figure 11d). At the optimum weight of g‐C_3_N_4_ in the hybrid, the UG‐50 photocatalyst showed dramatically enhanced photocatalytic activity for the HER, with a H_2_ evolution rate of 1411 µmol h^−1^ g^−1^, compared with UiO‐66 and g‐C_3_N_4_ (0 and 80 µmol h^−1^ g^−1^, respectively). These results confirmed that the annealing process promoted the formation of UiO‐66/g‐C_3_N_4_ heterojunctions and enhanced the charge separation efficiency in UG‐50, which enhanced the H_2_ evolution rate (more than 17‐fold). These results revealed the essential role of the UiO‐66/g‐C_3_N_4_ interfaces in enhancing the photocatalytic H_2_ production.

**Figure 12:**
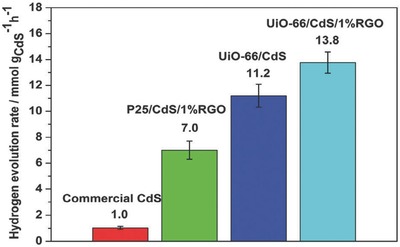
H_2_ evolution rates of various photocatalysts under visible light irradiation. Reproduced with permission.^164^ Copyright 2014, Royal Society of Chemistry.

Loading CdS with RGO is a useful method to reduce the possibility of electron‐hole pair recombination since graphene has high conductivity and superior electron mobility, which can accelerate the electron transfer in photocatalytic reaction.^166^ Lin et al. found that the hydrogen production performance of CdS was improved by the combined action of UiO‐66 and RGO due to the increased quantity of active sites and the minimized recombination of charge carriers.^164^ As a support, UiO‐66 was superior to inorganic semiconductor material. The UiO‐66/CdS/1% RGO photocatalyst displayed a very high photocatalytic HER rate, which was 13.8, 2 and 1.23 times greater than commercial CdS, P25/CdS/1% RGO and UiO‐66/CdS, respectively, as shown in **Figure** **12**.

In another study, highly porous Co_3_O_4_@carbon derived from ZIF‐67 MOFs was successfully embedded on the surface of BiVO_4_ nanosheets,^89^ in which the construction of a p‐n heterojunction between the porous Co_3_O_4_ (p‐Co_3_O_4_) and BiVO_4_ significantly promoted the charge transfer and reduced electron‐hole recombination. The p‐Co_3_O_4_@carbon/BiVO_4_ photoanode demonstrated ≈10 times higher photocurrent density than the bare BiVO_4_ and bulk Co_3_O_4_/BiVO_4_ for the OER. This system also demonstrated good stability for the OER without noticeable degradation after three recycled uses. More recently, a highly active Au@CdS/MIL‐101 heterostructure was successfully prepared.^165^ The MIL‐101(Cr) with large specific surface area was utilized as a support for well‐dispersed Au NPs, and CdS was selectively coated on the Au NPs.^165^ The good dispersion of CdS and Au NPs, as well as the strong surface plasmon resonance absorption of Au, led to enhanced electron separation and transfer. Under visible light irradiation, the Au@CdS/MIL‐101 heterostructure presented a high H_2_ production rate of 25,000 µmol h^−1^ g^−1^, which was 3.6 and 1.3 times greater than those of CdS and CdS/MIL‐101, respectively. These results suggested that the heterojunction in the photocatalysts played a critical role in the high photocatalytic activity, and these studies will encourage extensive research on heterostructured photocatalysts in the future.

Besides the separation efficiency of the photo‐induced charge carriers, the photocatalytic activity of the MOF‐based/derived photosensitizer/photocatalyst essentially relies on the excited state lifetime of the photo‐generated electron‐hole pair.^45^, ^87^, ^167^, ^168^, ^169^, ^170^, ^171^, ^172^, ^173^ For example, Lin and co‐workers synthesized a MOF based on {M[4,4′‐dcbpy]_2_bpy}^2+^ building blocks (where M = Ru or Os, bpy = 2,2′‐bipyridine and dcbpy = dicarboxy‐2,2′‐bipyridine), which can be readily excited to their long‐lived triplet metal‐to‐ligand charge transfer (^3^MLCT) states.^171^ The lifetimes of Ru^2+^ excited states in the Os‐doped MOF decreased progressively with increasing Os doping amount from 0.3 to 2.6 mol.%. The lifetime at 620 nm decreased from 171 ns in the pure Ru‐MOF to 29 ns in the sample with 2.6 mol.% Os doping. In the mixed‐metal samples, energy transfer was observed with an initial growth in Os emission corresponding with the decay rate of the Ru excited state. These results demonstrate rapid, efficient energy migration and long distance transfer in these isomorphous MOFs.

Morris and co‐workers have made outstanding contributions to the study of the lifetimes of the photo‐induced electron and holes in MOF‐based photocatalyst.^174^, ^175^, ^176^, ^177^ The effect of the doping amount of ruthenium(II) tris(5,5′‐dicarboxy‐2,2′‐bipyridine), Rudcbpy, on the lifetime of the emissive ^3^MLCT state of the Rudcbpy‐doped UiO‐67 MOF (Zr_6_(μ_3_‐O)_4_(μ_3_‐OH)_4_(bpdc)_6_ (bpdc = biphenyldicarboxylic acid)) was systematically investigated by Morris and co‐workers.^174^ They found that the lifetime of the emissive ^3^MLCT state corresponding to Rudcbpy centers incorporated into the MOF backbone was found to be sensitive to the Rudcbpy doping amount in the material. The excited state properties of the Rudcbpy‐doped UiO‐67 at low doping concentrations resembled that of Rudcbpy in dimethylformamide (DMF) displaying a long‐lived (≈1.4 µs) ^3^MLCT state. Increasing the Rudcbpy doping concentration in UiO‐67 was accompanied by a significant decrease in the emission lifetime, which was proposed to be due to the homogeneous energy transfer between the Rudcbpy centers.^174^, ^175^ Single Rudcbpy preferentially occupied the larger octahedral cages of UiO‐67 by incorporation into the backbone of the cage and experienced a DMF‐like solvation environment. At higher doping concentrations of Rudcbpy, in addition to incorporation of Rudcbpy into the backbone of the octahedral cavities, populations of encapsulated Rudcbpy were also found in separate octahedral UiO‐67 cavities. The decreased lifetime of the slow phase with increased doping concentration was attributed to the intermolecular energy transfer between neighboring Rudcbpy molecules incorporated into the backbone of the octahedral cages.

In another study, Morris and co‐workers found that this Rudcbpy‐doped UiO‐67 can be grown onto conductive fluorine‐doped tin oxide (FTO) coated glass substrates without changing its excited state properties or dynamics.^175^ The Rudcbpy dopant within the UiO‐67 films interacted with each other and underwent self‐quenching via a resonance energy transfer mechanism.^175^ The average distance between Rudcbpy was decreased in the film relative to similarly doped powders. This is attributed to an electrostatic effect upon formation of the framework due to increased charge at the bpdc self‐assembled monolayer on the surface of the substrate. These Rudcbpy‐doped UiO‐67 films can also be grown onto TiO_2_ as a sensitizing material for photovoltaic applications such as sensitized solar cells, which can broad the potential applications of MOFs in various solar energy‐based research fields.^177^

In addition, it was found that the coupling of nanosized carbon nitride nanosheets (CNNS) with UiO‐66 via a facile electrostatic self‐assembly method increased the lifetime of the photo‐generated electron‐hole pair of CNNS photocatalyst.^178^ It was demonstrated that the electrons from the photo‐excited CNNS can transfer to UiO‐66, which substantially suppressed the electron‐hole pair recombination in the CNNS, and also provided long‐lived electrons for the reduction of CO_2_ molecules that were adsorbed in UiO‐66. The calculated lifetime values were 481.4 ns for CNNS and 846.3 ns for UiO‐66/CNNS composite. As a result, the UiO‐66/CNNS photocatalyst exhibited a much higher photocatalytic activity than that of CNNS. This work highlights the synergistic incorporation of MOFs to C_3_N_4_‐based photocatalysts to increase the lifetime and improve the separation efficiency of the photo‐generated electron‐hole pair.

#### 3.2.4 A Brief Summary

The catalytic activities of some typical MOF‐based and MOF‐derived photocatalysts for the HER and OER are listed in **Table** **2**. In addition to the main active components, the activity of the photocatalysts for the HER and OER is heavily dependent on the sacrificial reagent and the co‐catalyst/active sites. Compared to MOF‐based electrocatalysts, in which the MOFs were more frequently used as precursors, MOFs have been widely used as photocatalysts directly, although they were often coupled with active sites/co‐catalysts to enhance their activity. The amine‐functionalized Ti‐MOF displayed superior photocatalytic activity for the HER due to the improved visible light response. The addition of metal/metal oxide improved the photocatalytic activity. However, the addition of an excessive amount of metal/metal oxide reduced the photoactivity for the HER and OER. For Pt, a loading amount of 1.5 wt.% in the Ti‐MOF‐NH_2_ photocatalyst resulted in the highest H_2_ evolution rate. Similarly, for CoO_x_ NP‐modified MOF, the optimum CoO_x_ amount of 3.9 wt.% was demonstrated for the OER, suggesting that the amount and the particle size of the co‐catalyst should be controlled and optimized to achieve high photocatalytic activity for the HER and OER. In addition to metals and metal oxides, functional materials, such as CdS, RGO, MoS_2_ and g‐C_3_N_4_, have been added to MOF to improve the photocatalytic activity for the HER. As shown in Table 2, MoS_2_ increased the photoactivity for the HER and was superior to Pt as a co‐catalyst.^130^ The H_2_ production rate of UiO‐66/CdS increased 25‐fold when 1.5 wt.% MoS_2_ was incorporated. The construction of dye‐sensitized systems can contribute to high photocatalytic activity; the selection of the dye, its amount and the incorporation method should be carefully investigated. In addition to the direct use of MOFs as photocatalysts, nanostructures and composites derived from MOFs were investigated as efficient photocatalysts for the HER and OER. Porous Co_3_O_4_ nanocages derived from PBA Co_3_[Co(CN)_6_]_2_ displayed high activity for the OER.^103^ However, the recycled performance of many MOF‐based photocatalysts must be enhanced. Additionally, in most of the studies summarized in this review, the quantum yield of the MOF‐based photocatalysts for water splitting was not presented, and the values that were presented are rather low and must be improved in the future.

**Table 2:** Photocatalytic activity of some typical MOF‐based and MOF‐derived photocatalysts for the HER and OER

No.	Photocatalyst	Target reaction	Sacrificial reagent	Production rate (µmol h^−1^ g^−1^)	Quantum yields (%)	Recycled times	Ref.
1	Bi‐based MOF	OER	AgNO_3_	≈180	/	/	162
2	Ti‐MOF	HER	Triethanolamine (TEOA)	0	/	/	142
3	Ti‐MOF‐NH_2_			≈170	/	/	
4	0.5 wt.% Pt/Ti‐MOF‐NH_2_			≈330	/	/	
5	1.5 wt.% Pt/Ti‐MOF‐NH_2_			≈500	≈1.3 at 420 nm	3	
6	2 wt.% Pt/Ti‐MOF‐NH_2_			≈460	/	/	
7	2.6 wt.% CoO_x_ NPs‐MIL‐101	OER	[Ru(bpy)_3_]^2+^–Na_2_S_2_O_8_	≈11,000	/	/	157
8	3.9 wt.% CoO_x_ NPs‐MIL‐101			≈15,000	/	2	
9	4.9 wt.% CoO_x_ NPs‐MIL‐101			≈13,000	/	/	
10	Small‐sized Ni NPs anchored in MOF‐5	HER	TEOA	3,022	7.8 at 520 nm	4	158
11	MnTD⊂MIL‐101	OER	Ceric ammonium nitrate	2,250	/	/	84
12	MIL‐101			125	/	/	
13	UiO‐66	HER	Na_2_S, Na_2_SO_3_	0	/	/	164
14	UiO‐66/CdS			1,700	/	6	
15	UiO‐66/CdS/1%RGO			2,100	/	/	
16	MoS_2_/UiO‐66/CdS	HER	Lactic acid	32,500	23.6 at 420 nm	4	143
17	UiO‐66/CdS			1,250	/	/	
18	g‐C_3_N_4_	HER	L‐ascorbic acid	80	/	/	163
19	UiO‐66			0	/	/	
20	g‐C_3_N_4_/UiO‐66 (1:1, w/w)			1,141	/	/	
21	Ti‐MOF‐Ru(tpy)_2_	HER	TEOA	≈200	0.2 at 500 nm	3	148
22	Pt complex immobilized MOF‐253	HER	CH_3_CN	≈58,000	1.63 at 440 nm	2	154
23	Pt@UiO‐66	HER	TEOA	3.9	/	/	149
24	2.54 mg g^−1^ of RhB/Pt@UiO‐66			5.6	/	/	
25	11.92 mg g^−1^ of RhB/Pt@UiO‐66			≈100	/	3	
26	ErB dye‐sensitized Pt/UiO‐66 octahedrons	HER	Methanol	460	0.25 at 420 nm	3	44
27	Hollow Fe_2_O_3_‐TiO_2_‐PtO_x_	HER	Lactic acid	1,100	/	5	145
28	Porous Co_3_O_4_ nanocages	OER	[Ru(bpy)_3_]^2+^–Na_2_S_2_O_8_	≈7,900	/	/	103
29	Porous Mn_x_Co_3−x_O_4_ nanocages			≈4,900	/	/	
30	Porous Fe_x_Co_3−x_O_4_ nanocages			4,000	/	/	
31	Co_3_O_4_/TiO_2_ p–n heterojunction	HER	Methanol	7,000	/	/	88

## 4 Challenges and Perspectives

In this review, the recent research progress of MOF‐based and MOF‐derived catalysts for electrocatalytic and photocatalytic water splitting reactions has been summarized. The applications of MOFs as direct catalysts and as precursors of catalysts for water splitting were included, and both the OER and HER were considered. MOFs have demonstrated promising applications in the field of electrocatalytic and photocatalytic water splitting for H_2_ production, and several impressive results are available. However, the field of MOF‐mediated water splitting is in its infancy, and remarkable enhancements are needed to make MOF‐mediated water splitting systems fully competitive. For example, the poor stability of MOF‐based catalysts in water is a major concern, which is attributed to the higher affinities of their ions for water molecules than the carboxylate ligands. The leaching of metallic and organic components from the MOFs into the reaction medium during the water splitting reactions is another major concern. The reaction mechanisms of MOF‐based catalysts for water splitting are still not well understood and require further investigation. The recycling of MOF‐based catalysts is also a major challenge due to the ultrafine size of most MOF‐based materials. The main challenges and perspectives for MOF‐based catalysts in electrocatalytic and photocatalytic water splitting reactions are discussed in the following sections.

### 4.1 MOF‐Based Catalysts for Electrocatalytic Water Splitting

There are three strategies to design MOF‐based electrocatalysts for electrocatalytic water splitting: morphology control/nanostructuring, constructing hybrids/composites and functional modifications. The morphology of the catalyst plays an important role in achieving high electrocatalytic activity. Electrocatalysts with special morphologies, such as CPHs and nano‐octahedrons, may show superior electrocatalytic activity than their related NPs. By tailoring the composition of the MOFs and the calcination temperature and atmosphere, various morphologies can be obtained with different catalytic properties for the water splitting reactions. For example, a MOF with a flower‐shaped morphology was developed with high electrocatalytic activity.^101^ However, due to the diversity of MOFs, it is hard to identify the most efficient morphology for the electrocatalytic OER/HER. A more systematic experimental investigation or theoretical study of the morphology‐activity relationship of MOFs is required.

In addition to the morphology, the interaction or synergistic effect between components in the electrocatalysts has a strong influence on the electrocatalytic activity of MOF‐derived materials and requires more attention in the development of MOF‐based and MOF‐derived electrocatalysts for the water splitting reactions. For MOF‐derived metal and metal oxide‐based catalysts, the leaching of metals during the operation is a major concern and could cause a serious decrease in the electrocatalytic activity and stability for water splitting in acidic or alkaline electrolytes. An effective solution to the deterioration is to encapsulate metal NPs into carbon nanotubes or carbon shells. The use of MOFs as precursors for the synthesis of core‐shell structured hybrid electrocatalysts may provide a new research direction for the development of MOF‐based catalysts for efficient water splitting. Despite the various MOF‐derived nanostructures, precise control of the morphology of these materials is absent because of the limited knowledge of the transformation process. A clearer understanding of this issue is urgently needed and would be helpful for the design and construction of fine nanostructures from MOFs with high surface areas, regular pores and tunable compositions for the electrochemical water splitting reactions.

Imidazolate linkers are often used as functional modifiers to improve the proton transfer process in the electrocatalytic water splitting reactions, which may be applied as a general method to design MOFs with improved charge transfer capability. In addition, functional carbon‐based materials, such as GO and rGO, can be used as modifiers for MOF‐based electrocatalysts to improve the charge transfer process. For example, CoP/rGO can be used as a bifunctional catalyst on both the anode and cathode for water splitting in alkaline solutions, with better activity (135 mV dec^−1^) than the integrated Pt/C and IrO_2_ catalyst couple (201 mV dec^−1^).^124^ However, the mechanism must be clarified. In addition, other carbon‐based materials, such as g‐C_3_N_4_, could be used to improve the charge transfer efficiency in the electrocatalytic OER and HER.

### 4.2 MOF‐Based Catalysts for Photocatalytic Water Splitting

As a photocatalyst for water splitting, the efficiency of a MOF‐based material is strongly affected by its sunlight absorption capability (band gap), the number of active sites and the charge separation/transfer efficiency. This review provided a summary of recent developments involving the use of MOFs as photocatalysts, with particular emphasis on several key strategies (band gap engineering, photosensitization, active site/co‐catalyst selection and optimization and the coupling of MOFs with other functional materials) to develop MOF‐based and MOF‐derived photocatalysts with high performance.

The band gap energy of MOFs can be tailored by changing the metal‐oxo clusters and bridging organic linkers. Several organic dyes have been used to sensitize MOF‐based photocatalysts, leading to significantly enhanced photoactivity under visible light irradiation. However, the stability of these organic‐linker‐modified MOFs and the organic dyes should be carefully considered in future research. MOFs themselves can also be used as photosensitizers for PEC water splitting and can significantly enhance the photoactivity of TiO_2_ under visible light.^139^ In the future, the match of the band structure of MOFs and TiO_2_ should be clarified by theoretical calculations, such as DFT, which may provide useful design guidelines for MOF‐based photosensitizers.

The incorporation of active sites, such as Pt NPs, into MOFs was found to dramatically enhance the photocatalytic activity.^142^, ^143^ However, the limited reserves and high cost of Pt hinder its large‐scare application. Recently, highly efficient, small‐sized Ni particles embedded in MOF‐5 as co‐catalysts/active sites with low overpotential for photocatalytic HER under visible light was demonstrated.^145^ A low overpotential of −0.37 V was obtained by this photocatalyst, which was comparable to that of Pt@MOF‐5. The development of non‐precious metal NPs in MOFs to enhance the photocatalytic activity requires more attention in the future. It is critical to develop MOF‐based photocatalysts that can be easily collected and reused. One solution is to incorporate magnetic materials into the MOFs. After water splitting, the magnetic photocatalyst can be easily collected by magnetic separation and recycled for further use. Magnetic MOF‐based composites should be next‐generation photocatalysts for water splitting.

The charge transfer/separation efficiency plays a vital role in the photocatalytic water splitting reactions. Various methods have been used to improve the charge transfer/separation efficiency. For example, electrons can be transferred from photoexcited organic linkers to metal‐clusters in Zr‐based MOFs (LLCT process).^149^ In the H_2_ generation process via the LLCT mechanism, the CB edge position of a titanium‐oxo cluster is more suitable for charge transfer since the CB potential of a titanium‐oxo cluster is more positive than that of its zirconium counterpart. Thus, Ti‐based MOFs are expected to be highly active photocatalysts for the HER. In addition to the design of new photocatalysts, functional additives, such as g‐C_3_N_4_, have been used to enhance the charge transfer/separation efficiency of MOF‐based photocatalysts. For example, g‐C_3_N_4_/UiO‐66 hybrid photocatalysts showed enhanced activity in the visible‐light‐driven HER, which was attributed to the efficient interfacial charge transfer from photoexcited g‐C_3_N_4_ to UiO‐66.^150^ These results demonstrated the potential use of MOFs and g‐C_3_N_4_ to construct active photocatalysts for water splitting. Based on the extensive studies of the application of MOFs in photocatalytic water splitting, the application of MOFs in solar cells will be a research focus in the future, although it is out of the scope of this article. It will be exciting to watch the rapid development of such new materials in the years to come.
